# Analyzing genomes: is there a duty to disclose?

**DOI:** 10.1186/1753-6561-6-S6-O14

**Published:** 2012-10-01

**Authors:** Amy McGuire

**Affiliations:** 1 Baylor College of Medicine, Houston, TX, USA

## 

The potential to discover unanticipated clinically significant genetic variants during the course of research has stimulated much debate about the obligations of researchers to communicate such findings to study participants. Published guidelines suggest that researchers have an obligation to communicate valid and significant results, especially if they are clinically actionable. I will present data on the practices and perspectives of investigators conducting genome-wide association studies (GWAS) and will caution against the establishment of a general obligation to return results in research. I will compare the role-specific responsibilities of researchers with those of clinicians and discuss emerging standards for genomic analysis and communication of test results in the clinical setting.

